# A Promising Approach in Home Visiting to Support Families Affected by Maternal Substance Use

**DOI:** 10.1007/s10995-020-03015-0

**Published:** 2020-11-27

**Authors:** Donna O’Malley, Danielle F. Chiang, Emily A. Siedlik, Katharine Ragon, Marcia Dutcher, Oneta Templeton

**Affiliations:** 1 Community Programs, Department of Social Work, Children’s Mercy Hospital, 2401 Gillham Road, Kansas City, MO 64108 USA; 2 Institute for Human Development, University of Missouri Kansas City, 215 W Pershing Road, Kansas City, MO 64108 USA

**Keywords:** Home visiting, Substance use, Parent–child interaction, Goal attainment

## Abstract

**Introduction:**

Many factors influence women’s use of alcohol and other drugs while pregnant and postpartum. Substance use impacts the maternal-child relationship during the critical neonatal period. The first days and months of human development lay the foundation for health and well-being across the lifespan, making this period an important window of opportunity to interrupt the transmission of trauma and stress to the next generation. Pregnant and postpartum women with a history of substance use require specialized support services.

**Methods:**

The Team for Infants Exposed to Substance abuse (TIES) Program provides a holistic, multi-disciplinary, community-based model to address the complex needs of families with young children affected by maternal substance use.

**Results:**

A multi-year implementation study of the model yielded results that indicate the effectiveness of this home-based family support intervention. The model focuses on reducing maternal alcohol and other drug use, increasing positive parenting, promoting child and maternal health, and improving family income and family housing. A key component of the model is establishing a mutual, trusting relationship between the home visiting specialists and the family. Foundational to the TIES model is a family-centered, culturally competent, trauma-informed approach that includes formal interagency community partnerships

**Discussion:**

This article describes elements of the model that lead to high retention and completion rates and family goal attainment for this unique population.

## Significance Statement

Prenatal and postpartum home visiting models are widely used to prevent child maltreatment, promote child-caregiver attachment, and foster positive parenting skills. Home visiting models target high-risk families, often with mental and behavioral health or substance use issues. Studies have shown that home visitors often feel ill-equipped to address the complex needs of families affected by substance use. This article describes a model developed specifically to provide specialized support to families affected by maternal substance use and presents data on family goal attainment.

## Introduction

During the twentieth century, infectious diseases were the main cause of childhood morbidity and mortality. Today, the social determinants of health and adverse childhood experiences are recognized as important predictors of health and well-being. Social determinants of health are defined as the conditions in which people are born, grow, live, work, and age (Marmot et al. 2008). The link between adverse childhood experiences and negative health outcomes is well established (Shonkoff et al. 2012). Home-based family support programs offer interventions that help create safe and healthy home environments for children at risk. Home-based family support programs are a key intervention to promote positive parenting and attachment, prevent child maltreatment, and facilitate linkage to community resources for high-risk families (Azzi-Lessing 2013). Home visiting models typically target pregnant and postpartum women with risk factors known to disrupt the parent–child relationship, such as history of trauma; intimate partner violence; mental health issues, including maternal depression; low academic achievement, often leading to low income; and a limited support network (Ammerman et al. 2015; Dauber et al. 2017a, b). Maternal substance use is another known risk factor for child maltreatment and may be a comorbidity among at-risk populations (Connelly et al. 2013; Dauber et al. 2017a, b; Michalopoulos et al. 2015). Few home visiting models, however, are equipped to address the complex needs of families affected by maternal substance use. Substance use among families receiving home visiting services has been associated with reduced program engagement and diminished outcomes (Azzi-Lessing 2013; Dauber et al. 2017a, b; Green et al. 2018). Many home visiting programs do not conduct systematic data collection related to substance use indicators, and few focus on substance use-related outcomes. In the 2016 Home Visiting Evidence of Effectiveness (HomVEE) review, nine of the 19 approved models collected substance-use outcomes data, and only three programs reported favorable substance-use outcomes (Novins et al. 2018).

Research findings indicate that even when maternal substance use is identified, home visitors report feeling ill-prepared to effectively respond to the needs of these mothers and their infants (S. Dauber et al. 2017a, b; Schreier et al. 2018; Tandon et al. 2008). Home visitors in these studies indicate a need for specialized training to address issues related to alcohol and other drug use (Schreier et al. 2018; Tandon et al. 2008). Home visitor education and training requirements vary widely across program models. Many programs employ paraprofessionals who lack the advanced training or clinical background required to successfully deliver therapeutic modalities addressing mental health and substance use issues (Azzi-Lessing 2013; Dauber et al. 2017a, b; Dauber et al. 2017a, b; Green et al. 2018; Novins et al. 2018). Home visitors serving these participants may also be exposed to high levels of stress and secondary trauma, which can lead to burnout and high turnover, potentially disrupting relationships between home visitors and families (S. Dauber et al. 2017a, b; Gill et al. 2007; Gomby 2007; Harden et al. 2010). These lessons learned from the field were used to develop the TIES promising approach.

### The TIES Model

**Fig. 1:**
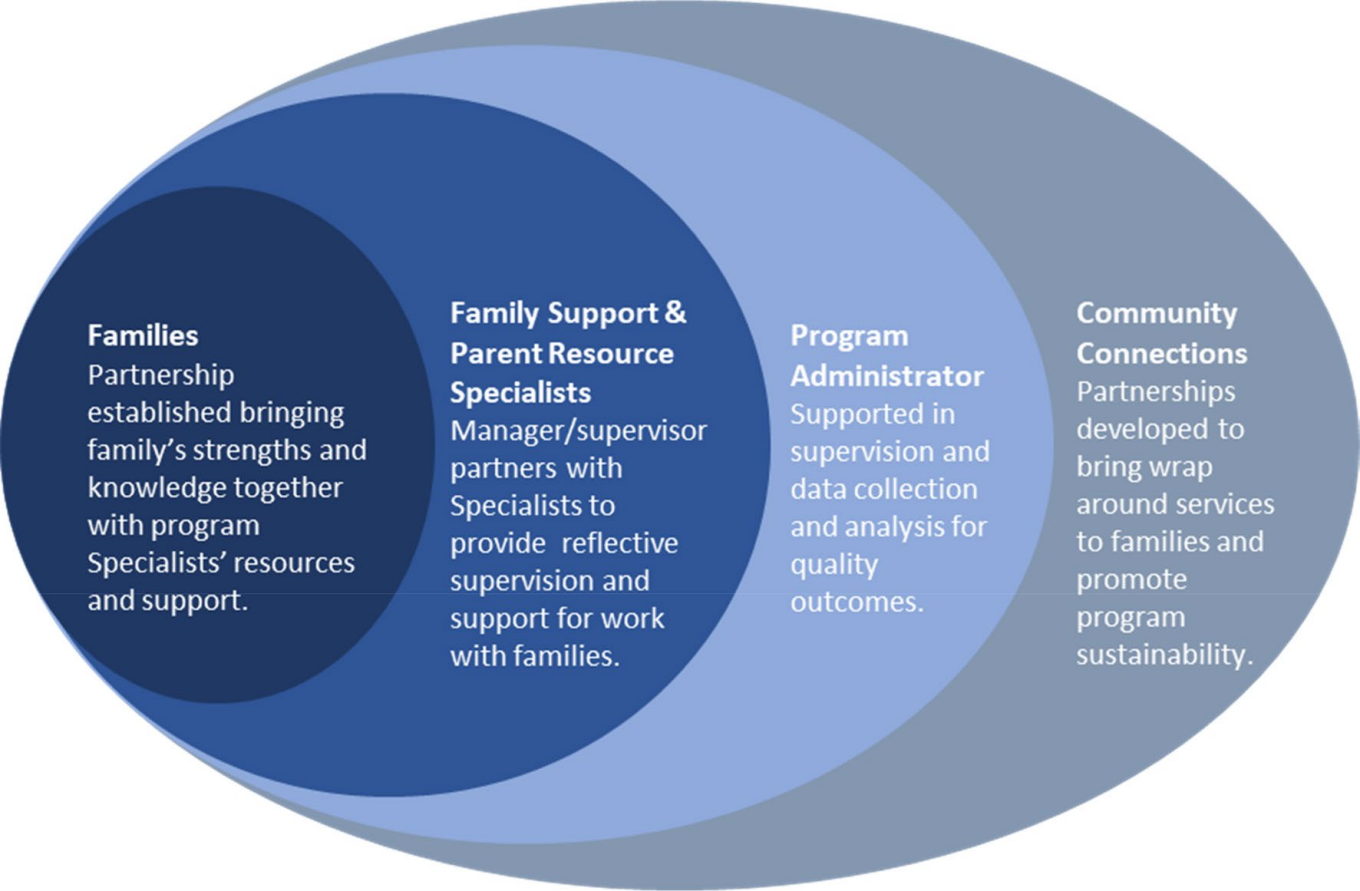
TIES social ecological model

The Team for Infants Exposed to Substance abuse (TIES) Program provides a holistic, multi-disciplinary, community-based model to address the unique needs of families affected by maternal substance use (Fig. 1). The TIES model, now in its 29th year, is delivered by master’s-prepared social workers along with endorsed infant family specialists to provide intensive, home-based services that enhance parent–child interaction, promote child development, and partner with families to set goals to encourage family stability. The design of the two-role model addresses gaps and challenges identified in other home visiting programs, including lack of specialized clinical preparation among program staff, high stress and turnover among staff, lack of a robust network of service providers for referral to care and treatment, and poor participant engagement. This model design has allowed the TIES Program to achieve significant positive outcomes in the domains of reduction in maternal substance use and increased positive parenting, child and maternal health, and family income and family housing. The effectiveness shown in goal attainment outcomes is attributed to the therapeutic relationship between the mothers and their specialists and the integrated community support, both hallmarks of the TIES Program. Due to these factors, the model was selected as a Promising Approach to be used by the Kansas Maternal, Infant, and Early Childhood Home Visiting (MIECHV) Program funded by US Department of Health and Human Services. Evidence-based status designation with the HomVEE project is also being pursued.

The professional team includes a master’s level Social Worker as the Family Support Specialist (FSS) and a Parent Resource Specialist (PRS) degreed in education, child development or related field. Each family has an FSS who meets with them weekly. The FSS provides direct services to families that include thorough and ongoing assessment, education and information, supportive counseling, and care coordination and goal setting with the family. The PRS is involved with each family as well to focus more specifically on the parent–child relationship offering information about child development, education and childcare systems, and parent assessment and coaching.

The PRS and FSS meet with each family jointly soon after enrollment to begin development of an individualized family plan. The PRS sees some families one-to-one regularly and others periodically depending on family needs, desires, capacity, and availability. Whether the PRS is seeing families individually or providing consultation and follow up with the FSS, the two specialists have distinct yet connected roles. They work as a team to best meet family needs and to maintain the voice of the child in the relationship. At the foundation of this collaboration is a strong, professional working relationship with mutual respect for the expertise of each.

There are two active sites and the model currently consists of six FSSs, two PRSs, one program coordinator who serves a small number of families, a data manager, and a program manager. Caseloads are limited to 10 families per FSS and 15 families per PRS. Staff provide direct services (e.g. counseling, crisis intervention, transportation, support for alcohol and other drug treatment, access to a women’s support group) and assistance in coordinating services with other community agencies (e.g. drug treatment, child welfare, health care, criminal justice). Women’s support groups for current participants and TIES alumni are typically held once or twice a month. The purpose of the support group is to model productive, healthy, mutual relationships among women. One monthly gathering provides an opportunity for participants to meet in a private location to give each other support as they engage in problem-solving dialogue. The second gathering provides families with a no- or low-cost family-oriented outing within the community. Four times per year, graduation celebrations are held for participants whose children have reached the age of 24 months.

This collaborative approach includes the mother as an important and equal partner in the 18- to 30-month journey to program completion and goal attainment. *(Note: the timeframe depends on when mom/baby is enrolled, prenatally or by 6 months of age.)* Team members receive extensive training in the principles of Trauma Informed Care. Training includes education and awareness of how a history of traumatic experiences impacts the health and well-being of participant and family and their capacity for relationships and how they bond with their baby. Sensitive practices are promoted to avoid retraumatizing clients, and self-care practices are taught and encouraged to strengthen participant’s and specialist’s own resilience. Creating a sense of safety and mutual trust empowers mothers to fully participate in making choices for their families throughout the program based on individual hopes and dreams, thus each participant’s course is customized to meet chosen goals. The relationship that develops between the mother and the TIES specialists provides a solid foundation for this shared journey. Past substance use histories and life challenges are acknowledged without judgment, and mothers are surrounded with the resources and support they need to succeed. This unique therapeutic relationship resets the maternal and child trajectory toward health and wellness.

The therapeutic partnership that supports positive behavior change begins with the knowledge that relationships are fundamental to all human development. The five guiding principles of the TIES Program honor the relationship of home visitor and participant in the context of a woman’s history and current hopes and dreams for herself and her family. These guiding principles allow each participant to navigate the stages of recovery as their capacity for growth and change allows. The five guiding principles are:

1. Women change in the context of relationships that recognize all their roles.
  Establishing a therapeutic relationship may be challenging.
  The relationship must be based on respect, empathy, and positive personal regard.
2. Families at risk experience compromised safety and security.
  Child safety is primary.
  Families’ survival needs must always be addressed.
3. Families have complex histories that include trauma and disadvantage.
  Trauma Informed Care approach is essential.
  Significant family pain and suffering may be present.
4. History and experience may have led to a sense of powerlessness.
  Focusing on maternal and family strengths restores a sense of control and confidence that builds hope.
  Parents are recognized as the most important resource for their children and as experts about their own family and its needs.
  Decision making and problem solving are facilitated through the partnership of home visitor specialists and the mother.
5. Readiness to change is expressed in a variety of ways.
  Individualized motivational strategies are required.
  Staff consistency, persistence, and accessibility are necessary.

### Home Visiting Specialists’ Experience and Skill Requirements

The TIES home visiting specialists are highly skilled in motivational interviewing techniques. They also screen for maternal depression and intimate partner violence and note the protective factors that are present. Specialists are required to address core competencies in training every year and to identify their individual training needs in supervision. All social work staff maintain professional licenses with the required training hours, and the PRSs secure and maintain endorsement through the Alliance for the Advancement of Infant Mental Health.

FSSs provide direct services to families that include thorough and ongoing assessment, education, counseling, and care coordination for the family. Each FSS has experience, expertise, and competency in effective interactions with families of diverse backgrounds. FSSs are trained in and demonstrate competence in knowledge of stages of recovery and related supports; identification of dually present mental health conditions particularly anxiety and depression; positive parenting skills to promote infant mental health and child development; and concepts of trauma informed care to support traumatized parents and build effective partnerships. They have a deep resource network to support coordinated community services and skills in navigating systems and linking to natural and family supports as well.

PRSs share many of the attributes of the FSSs, but they concentrate on the parenting and child development needs of the family. PRSs must be knowledgeable of typical and atypical child development, infant bonding and attachment, child guidance techniques, and the long- and short-term impact of parental substance use and other trauma on children. Additionally, PRSs require skills in relating with the families, promoting positive parent–child interaction, and sharing child guidance information in accessible formats. Their skills in communication and interaction with families allow them to share information with families in meaningful ways, such as adapting to their individual circumstances, building on the current activities for the household, and breaking down complex parenting tasks into smaller steps.

All home visiting specialists participate in both group and individual reflective supervision monthly. Reflective supervision is a collaborative relational interaction used to elicit cognitive and emotional understanding of thoughts and feelings related to working with traumatized individuals and populations. Routine reflective practice supported by experienced TIES Program leaders allows staff to grow in their capacity to explore and understand negative and difficult emotions that come from serving mothers with substance use. TIES staff benefit by building skills that allow them to maintain boundaries and manage their own reactions and emotions as they provide the intervention for the duration of the program until infants reach 24 months of age.

The Specialists’ experience, training, and access to reflective supervision to process challenging interpersonal work promotes staff retention. Participants benefit greatly from having a consistent relationship from enrollment to completion. Staff turnover in the TIES Program is extremely low with the seven current specialists and program coordinator having a total of 134 years in the program, and all but one having been with the TIES Program for at least seven years, for a mean of over 15 years in the program. This staff expertise and stability contribute to high retention of families, with a retention rate for those families eligible to have completed from 2013–2019 of 65%. Though the TIES Program works exclusively with families whose drug use makes them more guarded, more mobile, more likely to be incarcerated, and more concerned about risk to their parental custody, this retention rate is at the high end of home visiting programs in general (MIECHV Technical Assistance Coordinating Center 2015).

### Interagency Community of Support

Interagency partnerships are critical to building a network of resources that support maternal and infant health for families affected by maternal substance use. The TIES Program enjoys strong community support from agencies and organizations united by a common purpose to support families impacted by maternal substance use. Formed in 1990, the Community Programs Consortium oversees TIES Program operations and includes agency members who represent physical and mental health care, substance use treatment, child care and early intervention programs, intimate partner violence services, child protection and family court services, outreach and other social services. The Consortium meets bi-monthly and has been very effective in promoting coordination of services for families, sharing information about services and resources, identifying and addressing unmet needs, and planning strategies to address barriers and improve quality of services available in the community. Additionally, a Community Programs Advisory Council, made up of a diverse group of community advocates and program alumni, brings unique perspectives to the table. The Council meets quarterly, and the expertise of its members is relied on to review program evaluation data and information on services and resource needs, to provide community feedback about program perception and enhancements, and to promote sustainability. Activities include educating local, regional, and national legislators, finding and engaging with program funders, and strengthening linkages between the TIES Program and the greater community.

The integration of these components allows the TIES Program to address the gaps in specialized services for families affected by maternal substance use. This article describes the effectiveness of this model as demonstrated by positive participant outcomes.

## Methods

### Process

The TIES Program uses a multi-year strengths-based framework that facilitates strong therapeutic relationships between home visiting professionals and mothers and their families. The program provides social work and parent educator specialists to work with families in their homes to create a mutually designed plan that is both individualized and family oriented to promote overall physical, social, and emotional health.

Complex trauma histories are common in maternal and infant populations most impacted by substance use. Thus, the TIES model focuses intervention on multiple goal areas: reducing maternal substance use; building parenting skills and capacity to support child development; enhancing parent response to the child’s physical and behavioral health care needs; enhancing parent response to self-health/behavioral health care needs; improving access to stable income; and improving access to stable and safe housing. Goals are developed with families, and progress is tracked at five time points.

**Fig. 2:**
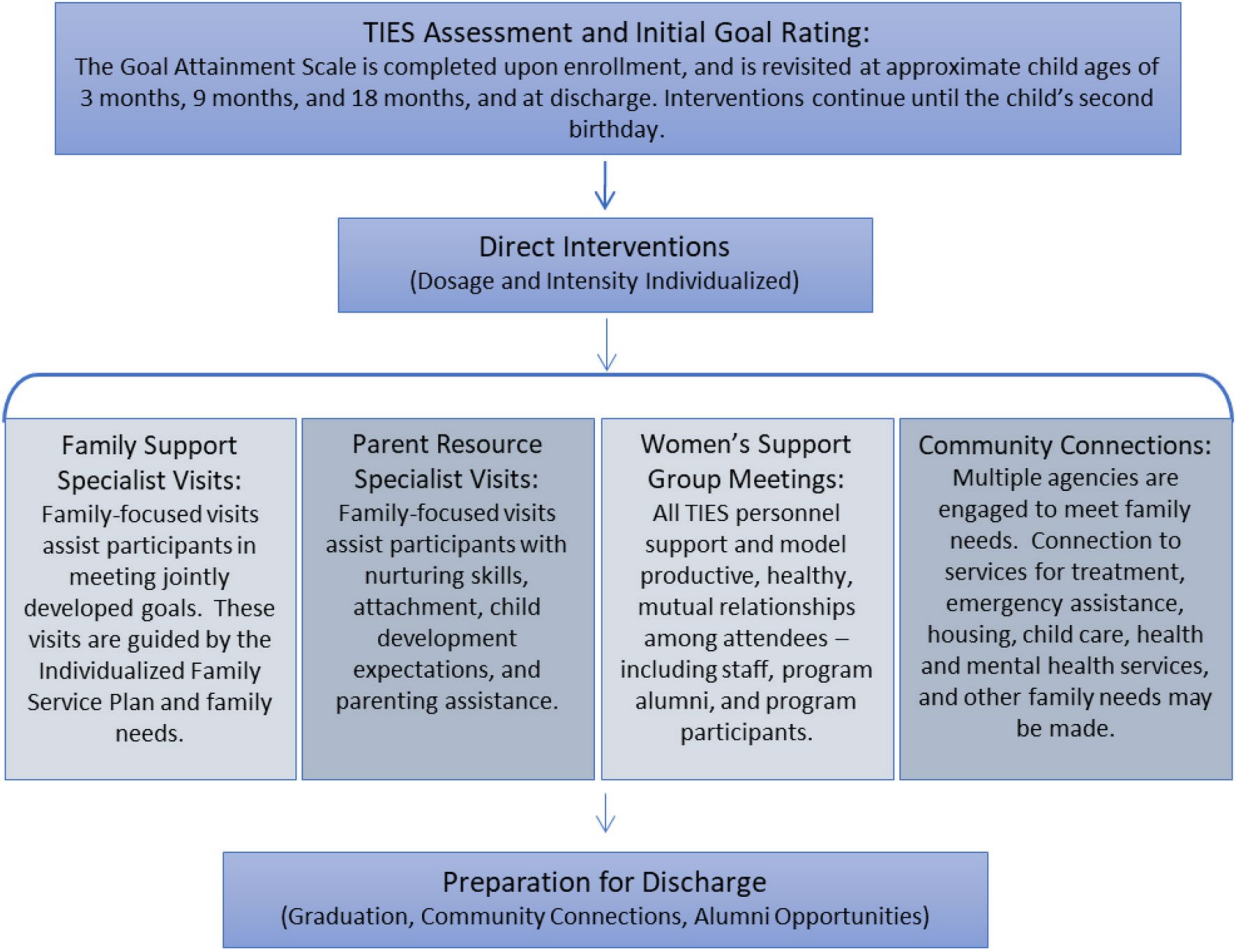
TIES process

Pregnant women and women with infants less than 6 months of age and their families who are affected by maternal substance use living in specific areas of the urban core of a large Midwestern city are eligible for the program. Participation is entirely voluntary and free of charge but is dependent upon a mother’s willingness to acknowledge that alcohol or other drug use is creating difficulties for her and her family and that she is interested in addressing those issues. Mothers must be at least 18 years of age and must have the infant in their custody or that of a relative to participate. The program lasts until the identified child reaches 24 months of age (Fig. 2).

Institutional Review Board (IRB) approval for program evaluation was secured through Children’s Mercy Hospital. A written consent form was presented to participants at the first visit, and formal consent was obtained before participants joined the program.

### Participants

Table 1 provides a description of the 220 families who participated in the TIES Program from 2012 through 2019. The majority of participants (56.4%) were White, non-Hispanic (87.7%) single moms (81.4%). A little over a third (39.1%) were between the ages of 25 and 29 years at enrollment, followed by 18 to 24 years old (27.7%) and 30 to 34 years old (21.4%). Nearly 41% of participants enrolled prenatally (40.5%), 43.2% enrolled postpartum when the child was less than 3 months old, and 16.4% enrolled postpartum when the child was greater than 3 but less than 6 months old. At enrollment, most participants were unemployed (84.1%) and had not completed high school (44.1%). The average monthly income for participants was $315. Over one-third of the participants (36.8%) rented/shared a home/apartment, and 30% lived with family/friends. For nearly 21% of moms, the index child was their only child, and 79.1% of participants had at least one additional child to whom the mother had access. Many participants used multiple substances, with 49.1% reporting cannabis, 36.4% reporting alcohol, 28.2% reporting amphetamines, and 21.4% reporting cocaine. Nearly 68% of participants also used tobacco products.

**Table 1:** Participant descriptive statistics

	*n*	% of Total
Enrollment group		
Prenatal	89	40.5%
Child < 3 Months	95	43.2%
Child 3–6 Months	36	16.4%
Age group		
18–24	61	27.7%
25–29	86	39.1%
30–34	47	21.4%
35–40	25	11.3%
41+	1	0.5%
Race		
African American	82	37.3%
American Indian/Alaska Native	3	1.4%
Asian	1	0.5%
Caucasian	124	56.4%
Multiracial	8	3.6%
Native Hawaiian/Pacific Islander	–	–
Other	2	0.9%
Ethnicity		
Hispanic/Latino	25	11.4%
Not Hispanic/Latino	193	87.7%
Not Provided	2	0.9%
Marital status		
Single	179	81.4%
Married	15	6.8%
Separated/divorced	16	7.2%
Domestic partner	5	2.3%
Common law	1	0.5%
Not provided	4	1.8%
Educational attainment		
Less than High School	97	44.1%
High School Diploma/GED	61	27.7%
More than High School	62	28.2%
Employment status		
Employed full time	13	5.9%
Employed part time	22	10.0%
Unemployed	185	84.1%
Additional children to whom mother has access		
0	46	20.9%
1–2	106	48.2%
3+	68	30.9%
Housing status		
Rents/Shares Own Home/Apartment	81	36.8%
Lives with Family/Friends	66	30.0%
Residential Treatment	9	4.1%
Shelter	12	5.5%
Supportive Housing	7	3.2%
Transitional Housing	36	16.4%
Homeless	81	3.6%
Correctional Facility	1	0.5%
Substance use type		
Alcoholic beverages (beer, wine, spirits, etc.)	80	36.4%
Amphetamine type stimulants (speed, diet pills, ecstasy, etc.)	62	28.2%
Cannabis (marijuana, pot, grass, hash, etc.)	108	49.1%
Cocaine (coke, crack, etc.)	47	21.4%
Hallucinogens (LSD, acid, mushrooms, PCP, Special K, etc.)	16	7.3%
Inhalants (nitrous, glue, petrol, paint thinner, etc.)	5	2.3%
Opioids (heroin, morphine, methadone, codeine, etc.)	24	10.9%
Sedatives or Sleeping Pills (Valium, Serepax, Rohypnol, etc.)	11	5.0%
Tobacco products (cigarettes, chewing tobacco, cigars, etc.)	149	67.7%
Mean Monthly Income (USD)	315	

### Measures

**Fig. 3:**
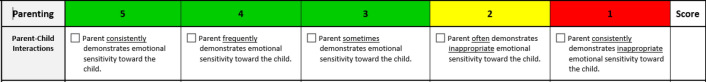
Goal attainment rubric excerpt—positive parenting: parent–child interactions

The TIES *Individualized Family Service Plan (IFSP)* contains a 5-point Likert scale created to assess and track participants’ goal attainment over time in the following areas: maternal substance use, positive parenting, child health, maternal health, family income, and family housing. Plans are specific to each family, and parents and specialists mutually agree on goals to be addressed at each review, based on individual needs of the family. The scale uses 1 to represent very low (crisis); 2 low (vulnerable); 3 adequate (stable); 4 high (advanced); and 5 very high (thriving) goal attainment. Each scale point is well defined in comprehensive rubrics specific to each goal area. For example, the positive parenting goal assesses basic needs, parent–child interactions, appropriate expectations, parenting strategies and problem-solving, access of resources and services, and safety and supervision. The Likert scale descriptors for a single component of positive parenting, parent–child interaction, is excerpted in Fig. 3. The tool also details the support and services the family will receive, including when, where, and how often the services will be delivered. Specific supportive activities are provided during home visits to increase participant knowledge, skills, abilities, and attitudes toward the chosen goal. Family specialists, together with parents, score the family’s status in goal areas on the Likert scale per rubric definitions at five time points. The score at intake (Time 1) serves as pre-test baseline and progress over time is charted at child’s age of 3–7 months (Time 2), 9–13 months (Time 3), 18–22 months (Time 4), and discharge (Time 5). The IFSP goal attainment scales have been validated by the external evaluator and determined to be reliable for this population.

### Analysis

Analysis of the entire set of longitudinal data (N = 220) began with descriptive statistics for each goal. We then employed one-way Repeated Measures Analysis of Variance (ANOVA) to examine the change over five time points in each of the six goal areas. In cases where we saw declines in mean goal scores, a separate paired sample t-test was used to determine if the mean score decline from one time point to the other was statistically significant. In addition, we also used paired sample t-tests to examine how soon participants started showing significant improvement on each goal. All analyses were conducted in SPSS (IBM Corp. 2019).

## Results

**Fig. 4:**
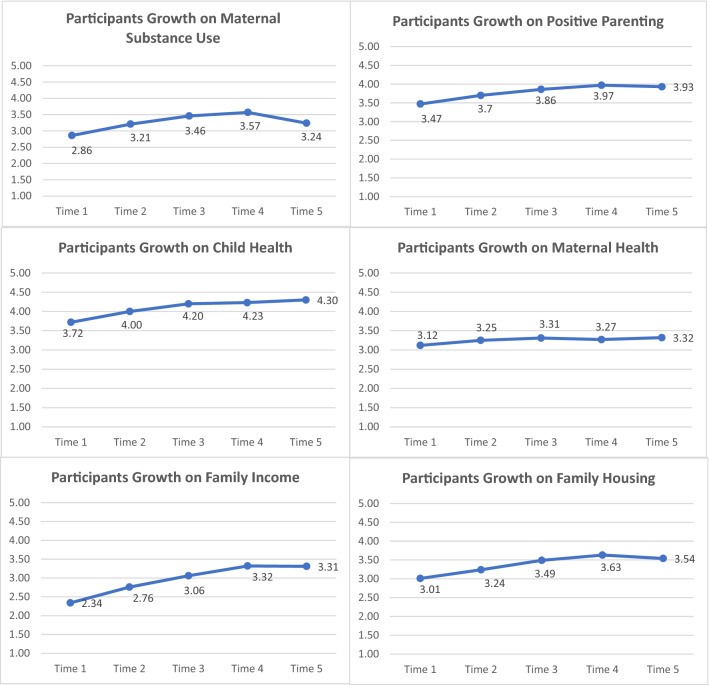
Mean goal attainment over time (N = 220). Time 1—intake, Time 2—child's age 3–7 months, Time 3—child's age 9–13 months, Time 4—child's age 18–22 months, Time 5—discharge

Findings demonstrate that TIES Program participation is positively correlated with goal attainment in multiple areas. The TIES model focuses on six primary goals: maternal substance use, positive parenting practices, positive child health outcomes, positive maternal health outcomes, family income and family housing. Figure 4 depicts the average goal scores of participants from intake to discharge. Mean scores in Fig. 4 resulted from descriptive statistics using the entire 220 families. As shown in Fig. 4, participants demonstrated a trend of improving in each goal area.

With significant Mauchly’s test of sphericity for each goal (see Table 2), the results in Table 3 from Repeated Measures ANOVA with a Greenhouse–Geisser correction confirmed the trends shown in Fig. 4. Specifically, results showed a significant mean score increase in reduced maternal substance use for women as they proceeded in the program, *F* (2.59, 241.24) = 8.88, *p* < .001, *ŋ*_*p*_^*2*^ = .09. Although there was a dip in reduced maternal substance use from Time 4 to Time 5 as seen in Fig. 4, a separate paired samples t-test using the entire sample indicated that the decline was not statistically significant, *t* (99) = .40, *p* = .69. Positive parenting scores improved significantly over time, *F* (2.68, 168.50) = 29.61, *p* < .001, *ŋ*_*p*_^*2*^ = .32. As seen in Fig. 4, there was a slight decline on positive parenting from Time 4 to Time 5, however, a paired samples t-test results indicated that the decline was not statistically significant, *t* (87) = .26, *p* = .79. For goals related to child and maternal health, results showed that the mean goal scores significantly increased for children over time, *F* (2.82, 169.03) = 11.31, *p* < .001, *ŋ*_*p*_^*2*^ = .16, although not for women, *F* (2.71, 238.19) = .51, *p* = .66, *ŋ*_*p*_^*2*^ = .01. However, a separate paired samples t-test revealed that the maternal health goal was significantly improved from intake to discharge, *t* (109) = ‒2.14, *p* = .036. Finally, participants had a significant improvement in mean scores in family income, *F* (2.81, 261.72) = 35.75, *p* < .001, *ŋ*_*p*_^*2*^ = .28, and in family housing, *F* (3.16, 297.39) = 14.60, *p* < .001, *ŋ*_*p*_^*2*^ = .13. A separate paired samples t-test indicated that the slight decline from Time 4 to Time 5 on family housing was not significant, *t* (99) = ‒ .46, *p* = .65. Post hoc pairwise comparison tests using the Bonferroni correction (see Table 4) revealed that mean goal scores significantly increased from intake to 3–7 months, continued to 9–13 months, to 18–22 months and to discharge in four out of six goals (maternal substance use, child health, family income, and family housing). Positive parenting mean scores significantly improved from intake to 9–13 months, continued to 18–22 months and discharge.

**Table 2:** Mauchly’s test of sphericity for repeated measures ANOVA

Goals	*W*	*χ*^2^	*df*	*p*
Maternal substance use	0.41	81.24	9	< .001
Positive parenting	0.34	65.70	9	< .001
Child health	0.43	48.72	9	< .001
Maternal health	0.41	77.45	9	< .001
Family income	0.45	73.08	9	< .001
Family housing	0.63	43.34	9	< .001

**Table 3:** Summary of repeated measures ANOVA with Greenhouse–Geisser correction

Goals	*df*	*MS*	*F*	*p*	*ŋ*_*p*_^*2*^
Maternal substance use (N = 94)	2.59	8.27	8.88	< .001	0.09
Error	241.24	0.93			
Positive parenting (N = 64)	2.68	16.19	29.61	< .001	0.32
Error	168.50	0.55			
Child health (N = 61)	2.82	3.61	11.31	< .001	0.16
Error	169.03	0.32			
Maternal health (N = 89)	2.71	0.65	0.51	0.66	0.01
Error	238.19	1.26			
Family income (N = 94)	2.81	26.54	35.75	< .001	0.28
Error	261.72	0.74			
Family housing (N = 95)	3.16	9.29	14.6	< .001	0.13
Error	297.39	0.64			

*Note* *MS *mean squares, effect size = partial η^2^

**Table 4:** Significant Mean Difference t-tests (all significant at p < .05)

Goal	Compare	Mean	SE	p	95% CI	
		Difference			Lower	Upper
Maternal substance use (N = 94)	T1–T2	0.34	0.09	< .001	0.09	0.59
	T1–T3	0.57	0.10	< .001	0.29	0.85
	T1–T4	0.55	0.13	< .001	0.17	0.92
	T1–T5	0.52	0.14	< .001	0.12	0.93
Positive parenting (N = 64)	T1–T3	0.46	0.08	< .001	0.22	0.69
	T1–T4	0.62	0.09	< .001	0.35	0.90
	T1–T5	0.59	0.11	< .001	0.28	0.90
	T2–T3	0.77	0.12	< .001	0.42	1.12
	T2–T4	0.94	0.13	< .001	0.57	1.30
	T2–T5	0.91	0.14	< .001	0.49	1.33
Child health (N = 61)	T1–T2	0.23	0.07	0.03	0.02	0.44
	T1–T3	0.43	0.10	< .001	0.13	0.73
	T1–T4	0.45	0.11	< .001	0.13	0.76
	T1–T5	0.49	0.10	< .001	0.18	0.80
	T2–T5	0.26	0.08	0.02	0.03	0.49
Family income (N = 94)	T1–T2	0.55	0.09	< .001	0.29	0.82
	T1–T3	0.91	0.11	< .001	0.60	1.22
	T1–T4	1.05	0.11	< .001	0.73	1.37
	T1–T5	1.03	0.14	< .001	0.64	1.44
	T2–T3	0.36	0.08	< .001	0.13	0.58
	T2–T4	0.50	0.11	< .001	0.20	0.80
	T2–T5	0.49	0.12	< .001	0.13	0.84
Family housing (N = 95)	T1–T2	0.34	0.08	< .001	0.12	0.56
	T1–T3	0.54	0.10	< .001	0.25	0.82
	T1–T4	0.64	0.10	< .001	0.36	0.92
	T1–T5	0.68	0.13	< .001	0.32	1.04
	T2–T4	0.30	0.10	0.04	0.01	0.60

*T1* Intake, *T2* Child's Age 3–7 Months, *T3* Child's Age 9–13 Months, *T4* Child's Age 18–22 Months, *T5* Discharge

Furthermore, paired-samples t-tests (N = 220) were conducted to examine how early participants showed significant gains staying in TIES Program even if they didn’t complete the program or missed data points. Results indicated that there was a statistically significant improvement from intake to 3–7 months in five out of six goals: maternal substance use, *t*(166) = ‒ 5.29, *p* < .001; positive parenting, *t*(126) = 2.58, *p* = .011; child health, *t*(119) = ‒ 4.13, *p* < .001; family income, *t*(164) = ‒ 6.91, *p* < .001; and family housing, *t*(166) = ‒ 4.66, *p* < .001. The maternal health goal showed a significant improvement from intake to discharge, *t*(109) = ‒ 2.13, *p* = .036. Therefore, we can conclude that participants in the TIES Program grew consistently over time, sustained their gains during participation, and benefitted even when participation was as brief as three to seven months. Statistically significant growth in participant outcomes provides evidence of the effectiveness of the TIES model.

## Discussion

### Promising Results of the TIES Model

The model has demonstrated promising and encouraging results in that overall families demonstrated notable growth in all six goals over the course of the intervention. Although faster improvement was noted in maternal substance use, positive parenting, child health, family income, and family housing, as compared to maternal health, five of six goals showed statistically significant improvement. Furthermore, the decrease from Time 4 to Time 5 in reduced maternal substance use, positive parenting, and family housing was within the standard deviation, with the largest magnitude of dipping of .33, and is therefore negligible. Even with the decrease in mean scores at Time 5, participants still managed to stay above the adequate (stable) level of goal attainment in those areas. Given the multiple challenges TIES families face, to achieve a stable outcome across multiple domains is indicative of significant success. Decreases in mean scores from Time 4 to Time 5 may be due to participants adjusting to the conclusion of the TIES Program and equipping themselves to navigate life without TIES supports, and the potential stress that may cause. Declining scores across these time points may also be attributed to the developmental stages of children at 18–24 months, and the new challenges presented when parenting mobile, verbal children who are learning to assert themselves and gaining independence.

A growing body of literature indicates the adoption of negative health behaviors related to drug use and other addictions often has root causes in adversities experienced in early childhood (Felitti et al. 1998). What health care providers and other professionals see as the problem, maternal substance use, is often a behavior that has been adopted by a woman to cope with a significant trauma history. At enrollment, a typical TIES participant is pregnant or recently postpartum, lives in poverty, is unemployed, may be homeless, has less than a high school degree, and lacks adequate resources to care for herself or her children. Many participants have had previous interactions with law enforcement, child welfare, and the court system. Few participants have experienced a trauma-informed, culturally sensitive intervention intentionally designed to address their drug use and promote their physical, mental, and emotional health and well-being and that of their baby. The TIES model demonstrates the importance of meeting mothers where they are in their lives. Designed as a trauma informed intervention that offers mothers respect, connection to resources, and flexibility in managing the demands of motherhood while in recovery, the TIES Program provides a path toward resilience, recovery, and healing.

### Limitations and Future Research

Participant characteristics and program features could be included in the statistical model to help explain how participants’ various backgrounds and situations might have affected goal attainment.

To further demonstrate the TIES Program’s effectiveness, major effort and emphasis is now allocated to recruiting a control group of participants and collecting comparative data. This data will be included in future research and evaluation and pursuit of evidence-based model designation for the TIES Program. In addition, opportunities to replicate the TIES Program in other communities would be highly desirable.

Future investigation may also focus on exploring the factors that impact various growth rates for participants, and the interaction effects among the six goals that contribute to participants’ growth. For example, preliminary findings show that the growth rate for maternal substance use and family housing synchronized, as did the growth rate for positive parenting and child health. More specific hypotheses could be explored and investigated in future studies including how the role of staff retention and inter-agency community support impact participants’ overall success.

### Implications/Conclusion

The prenatal and postpartum period is critical to optimal human development. Mothers with substance use often have extensive trauma histories. The TIES Program is designed to interrupt the intergenerational transfer of trauma and toxic stress from substance-using mothers to their newborns. Enriched early life mother/baby relationships and safe home environments are known to play a powerful role in setting a trajectory toward positive physical and mental health across the lifespan. Interventions in the TIES model focus on modifiable factors related to trauma and disadvantage which are prevalent in the family histories of participants (Traub and Boynton-Jarrett 2017). This two-role model integrates the expertise of a masters level social worker focused on building a strong therapeutic home-based family support specialist/participant relationship with an equally expert parenting specialist focused on the mother/baby relationship. This innovative approach supports the unique needs of mothers in recovery and the critical mother/baby relationship simultaneously. The TIES Program aims to address root causes of health inequity that often lead to poor physical and mental health outcomes and substance use. The retention and completion rates of TIES participants are the result of a highly skilled social work and parenting provider team, strong community partnerships, well-vetted resources, mutually valued therapeutic relationships of mothers/providers, and enhanced parenting support. Just as physical health needs sometimes require an accurate dose of a specific prescription medication to help individuals heal, TIES participants require an accurate and specific dose of supportive intervention delivered at the right time, in the right place, by the right method to support their recovery and healing. Every TIES home visit is an opportunity to deliver a dose of supportive intervention and encouragement. Participants and their TIES specialists share a belief that success is possible.
